# Sub-retinal pigment epithelium tubules in non-neovascular age-related macular degeneration

**DOI:** 10.1038/s41598-022-19193-6

**Published:** 2022-09-07

**Authors:** Serena Fragiotta, Mariacristina Parravano, Riccardo Sacconi, Eliana Costanzo, Daniele De Geronimo, Francesco Prascina, Vittorio Capuano, Eric H. Souied, Ian C. Han, Robert Mullins, Giuseppe Querques

**Affiliations:** 1 UniCamillus-Saint Camillus International University of Health Sciences, Rome, Italy; 2 IRCCS-Fondazione Bietti, Rome, Italy; 3 Department of Ophthalmology, IRCCS Ospedale San Raffaele, University Vita-Salute, Via Olgettina, 60, 20132 Milan, Italy; 4 Centre Hospitalier Intercommunal de Creteil, Creteil, France; 5 The Institute for Vision Research, Department of Ophthalmology and Visual Sciences, Carver College of Medicine, University of Iowa, Iowa City, IA USA

**Keywords:** Visual system, Retina

## Abstract

To describe a novel optical coherence tomography (OCT) signature resembling sub-retinal pigment epithelium (RPE) tubules (SRT) in non-neovascular age-related macular degeneration (AMD). Patients suffering from non-neovascular AMD with complete medical records and multimodal imaging were retrospectively revised in three different tertiary care centers. Multimodal imaging included color fundus photograph, spectral-domain OCT (Spectralis, Heidelberg Engineering, Germany), fundus autofluorescence, OCT angiography (RTVue XR Avanti, Optovue, Inc., Fremont, CA). A total of 7 eyes of 7 patients with drusenoid pigment epithelium detachment (PED) were consecutively analyzed. The sub-RPE tubules appeared as ovoidal structures with a hyperreflective contour and hyporeflective interior appreciable in the sub-RPE-basal lamina (BL) space on OCT B-scan. The anatomical location of the sub-RPE formations was lying above the Bruch’s membrane in 5/7 cases (71.4%) or floating in the sub-RPE-BL space in 2/7 cases (28.6%). En-face OCTA revealed a curvilinear tubulation-like structure corresponding to SRT without flow signal. Sub-RPE tubules represent a newly identified OCT signature observed in eyes with drusenoid PED. The presumed origin may include a variant of calcified structure or alternatively activated RPE cells with some residual BL or basal laminar deposits attracted to BrM for craving oxygen.

## Introduction

Advances in multimodal imaging and histopathological correlations have allowed identifying distinctive signatures and high-risk features associated with age-related macular degeneration (AMD) progression^1–3^. In particular, outer retinal tubulations (ORT) have been recognized as ovoidal hyperreflective structures surrounding a hyporeflective core located in the outer nuclear layer (ONL) in correspondence to atrophic regions^4–6^.

ORT can be distinguished from a cystic structure containing fluid (i.e., macular edema) by the presence of a hyperreflective border, the refractoriness to anti-vascular endothelial growth factor therapy (anti-VEGF), the relatively unchanged structural appearance with time, and the association with retinal pigment epithelium (RPE) atrophy or fibrosis^4,5,7,8^.

Histopathological correlations have demonstrated that ORT is delimited by an external limiting membrane (ELM) border, including photoreceptors pointing towards the lumen and a degenerated or absent RPE^5^. Müller cells represent one of the primary cells involved in ORT formation, sealing off photoreceptors from the RPE-basal lamina (BL)-Bruch’s membrane (BrM), and configuring the end-stage ORT formed by Müller cells without photoreceptors^5,9^. It has been hypothesized that Müller cells respond to an RPE injury, becoming reactive and upregulating the fibrillary acidic protein expression. Therefore, in the end-stage ORT with an absent RPE and degenerated photoreceptors, Müller cells activated to reinforce the ELM border^5,9,10^.

The present study aimed to describe a novel signature resembling a tubulation located in the sub-RPE space in non-neovascular AMD. This study provides a clinicopathological sample describing a potential histological interpretation for the multimodal findings presented. Based on the clinical and multimodal imaging features, the term "sub-RPE tubules (SRT)" was coined to describe this signature. Pathogenetic hypotheses, clinical features, and potential prognostic implications were explored.

## Methods

A retrospective observational cross-sectional study was performed revising the medical record and multimodal imaging findings on over a thousand non-neovascular AMD cases at three different tertiary care centers IRCCS San Raffaele Hospital, University Vita-Salute (Milan, Italy), IRCCS-Bietti Foundation (Rome, Italy), and Centre Hospitalier Intercommunal De Creteil (Creteil, France). This study adhered to the Declaration of Helsinki tenets and was approved by the Institutional Review Boards (IRBs) at the Central Ethics Committee—IRCCS Lazio (Section IFO—Bietti Foundation), Rome, Italy. All patients gave their written consent before inclusion.

Patients with non-neovascular AMD were considered only if they had a comprehensive ophthalmological examination and at least 3 of the following multimodal imaging modalities: color fundus photograph (CFP), MultiColor^®^ (MC), near-infrared reflectance (NIR), optical coherence tomography (OCT), OCT angiography (OCTA), fundus autofluorescence (FAF), fluorescein angiography (FA), and indocyanine green angiography (ICGA). Exclusion criteria comprised outer retina and RPE atrophy (cRORA)^11^ involving the foveal center at baseline, any form of macular neovascularization (MNV), severe ocular media opacities, and other ophthalmic comorbidities.

### In vivo multimodal imaging analysis

Spectral-domain optical coherence tomography (SD-OCT, HRA2 + OCT, Heidelberg Engineering, Heidelberg, Germany) was acquired with a minimum acquisition protocol of 20- × 15-degree pattern centered on the fovea constituting of 19 OCT B-scans. FAF and Multicolor^®^ were acquired simultaneously with the same instrumentation. Color fundus photograph (CFP) was obtained with either Clarus 500 (Carl Zeiss Meditec, Version 01 05/2017) or Topcon TRC-50DX (Topcon fundus camera, Tokyo, Japan). FA, ICGA, and/or OCTA were obtained to exclude the presence of any MNV subtypes. OCTA was achieved using either RTVue XR (RTVue XR Avanti, Optovue, Inc., Fremont, CA) equipped with the AngioVue software (version 2017.1.0.151; Optovue Inc) or Spectralis OCT (Heidelberg Engineering, Heidelberg, Germany). A 3- × 3-mm or 6- × 6-mm volumetric scan pattern was considered with projection artifact removal. The signal strength cut-off was set ≥ 45 signal strength index (SSI) for RTVue and > 15 Q score for Spectralis OCT.

### Multimodal imaging features

Pigment epithelial detachment (PED) was defined as a pale or white mound RPE elevation measuring at least 350 µm in the narrowest diameter calculated between edges on OCT B-scan^12,13^. In the drusenoid PED, the RPE is separated from BrM and is associated with either a BL or basal laminar deposits (BLamD) of variable thickness adhering to the RPE basement membrane. Therefore, we refer to the RPE band in the context of a PED as RPE + BL^14,15^. cRORA was defined according to the Classification of Atrophy (CAM) Report 3^11^.

ORTs were circular or ovoidal in shape, delimited by a hyperreflective border with an hyporeflective interior in the context of the ONL layer^4^. An open ORT consisted of a hyperreflective band without a complete connection to the outer portion of the hyporeflective lumen, while a closed ORT indicated a completely closed hyperreflective band around the central hyporeflective lumen^9^.

Calcified nodules appear as heterogeneous internal reflectivity within drusen (HIRD) revealed on OCT, and glistening appearance on color fundus photography (CFP)^2^. Hyperreflective crystalline deposits (HCD) were diagnosed on OCT as single or multiple highly reflective lines in the sub-RPE-basal laminar space^16,17^.

Plateau is a descriptive term originally described as a “wedge-shaped subretinal hyporeflectivity”^15,18^. This signature represented a residual structure seen on OCT after RPE loss, visible as a thin hyperreflective surface (contouring the RPE border of a former PED) with a heterogenous interior characterized by a reduced internal reflectivity usually accompanied by small hyperreflective foci (HF)^15^.

## Results

A total of 7 cases (7 females) with SRT were identified over 138 patients with drusenoid PED consecutively analyzed, exhibiting a prevalence of 5.07%. Mean age was 75 (range 60–87 years). BCVA was 0.30 LogMAR (range 0.09–0.49 LogMAR, Snellen equivalent 20/40).

**Figure 1:**
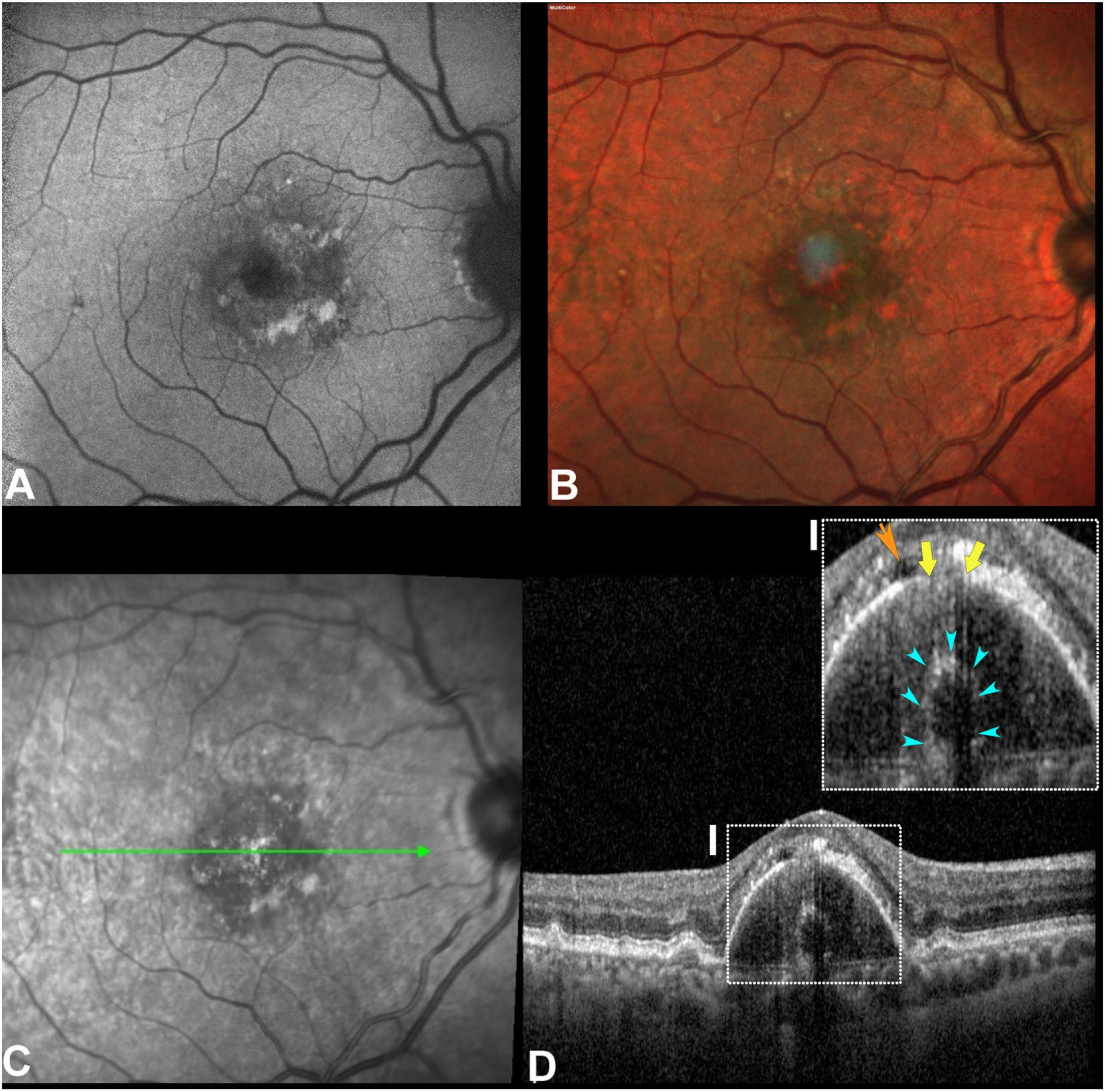
Multimodal imaging of a sub-retinal pigment epithelium (RPE) tubule. (**A**) Fundus autofluorescence; (**B**) MultiColor image; (**C**) Near-infrared reflectance (NIR), the green line represented the optical coherence optical tomography (OCT) scan point; (**D**) OCT b-scan demonstrates a dome-shaped RPE detachment (PED) with an ovoidal hyperreflective structure with an hyporeflective content apparently located at the inner collagenous layer of the Bruch's membrane. Teal arrowheads demarcate this structure (magnified inset I), the overlying RPE (yellow arrows) presents a localized defect similar to an RPE aperture. In the sub-retinal space, a minimal hyporeflective space (orange arrowheads) can be appreciated with an RPE swarm.

Sub-RPE-BL tubules were recognized on OCT B-scan as a solitary ovoidal sub-RPE-BL or BLamD structure with hyporeflective lumen and delineated by a hyperreflective wall evident in all the cases (Fig. 1).

**Figure 2:**
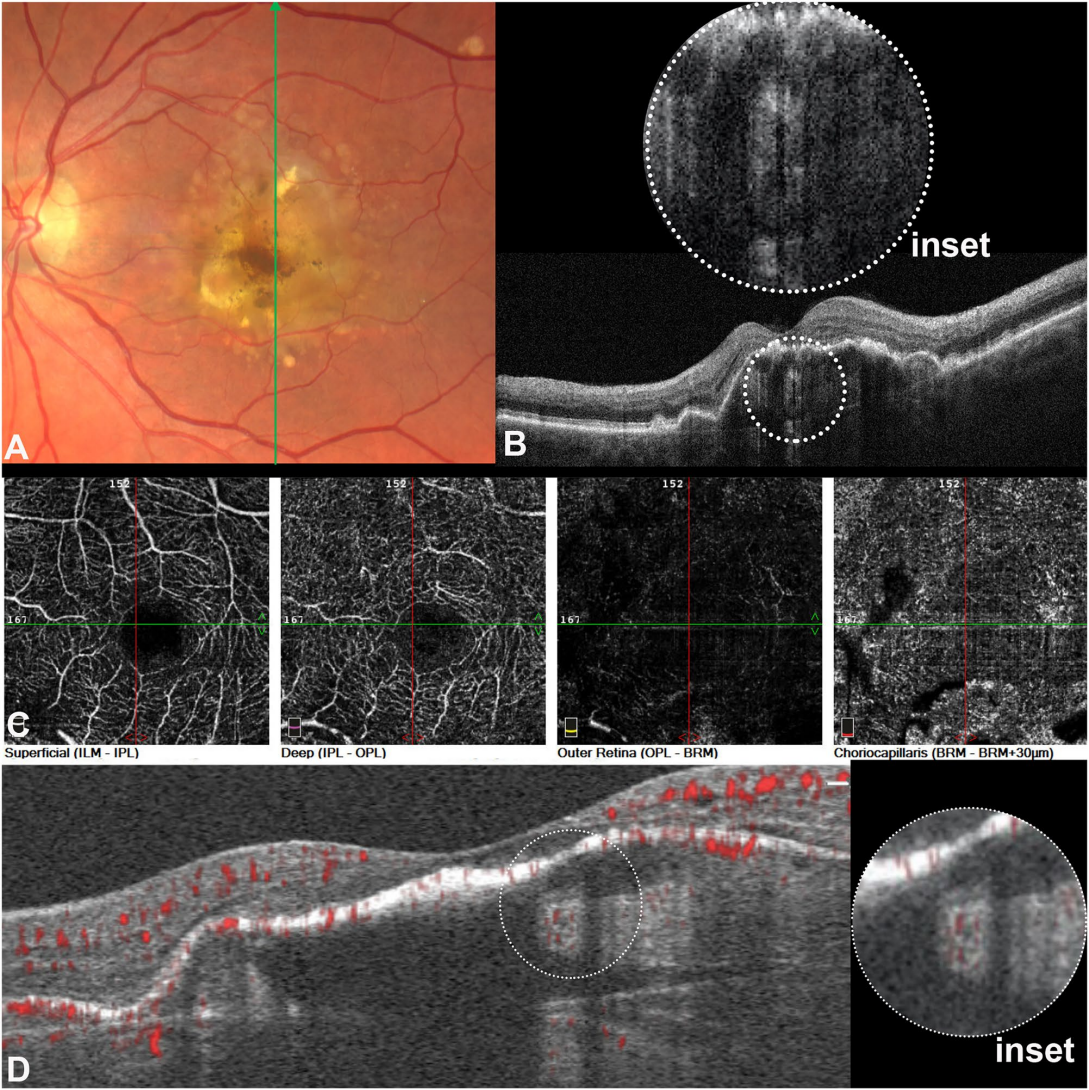
Sub-retinal pigment epithelium (RPE) tubule (SRT) features. (**A**) Color fundus photograph showing a large drusenoid pigment epithelium detachment (PED) with pigmentary changes in absence of glistening. (**B**) Optical coherence tomography (OCT) B-scan demonstrating the SRT as an ovoidal structure with hyperreflective border and an hypoereflective center (inset), the lesion appears separated from Bruch’s membrane, (**C**) Enface OCT angiography demonstrating the absence of flow alterations in correspondence of the lesion in the different vascular slabs. (**D**) OCTA b-scan demonstrating no evident flow signal within the lesion, better seen on magnification.

Multimodal characteristics at baseline are summarized in Table 1. On CFP or MC, no evidence of glistening was noted at baseline in all the cases available (Fig. 2). FAF appeared iso- or hypo-autofluorescent in correspondence of the lesions, as detailed in Table 1. The NIR presentation was heterogeneous, but mostly hyporeflective in 3/5 of cases.

**Table 1:** Multimodal features of sub-retinal pigment epithelium tubules (SRT) at baseline.

	N	Features	N/tot (%)
CFP	5/7	No glistening	5/5 (100%)
FAF	5/7	Hypoautofluorescent	3/5 (60%)
		Isoautofluorescent	2/5 (40%)
NIR	5/7	Hyporeflective	3/5 (60%)
		Isoreflective	1/5 (20%)
		Hyperreflective	1/5 (20%)
**OCT**	7/7		
HRF		Presence of intraretinal HRF	4/7 (57.14%)
RPE		RPE discontinuity	2/7 (28.57%)
HTM		Presence of HTM	3/7 (42.85%)
OCTA	5/7	Absence of flow signal	5/5 (100%)

*N* number of cases available for each imaging modality, *CFP* color fundus photograph, *FAF* Fundus autofluorescence, *NIR* near-infrared reflectance, *OCT* optical coherence tomography, *HRF* Hyperreflective foci, *RPE* retinal pigment epithelium, *HTM* hypertransmission, *OCTA* optical coherence tomography angiography, *RPE* discontinuity is intended as focal disruptions or breaks in the continuity of the RPE band.

**Figure 3:**
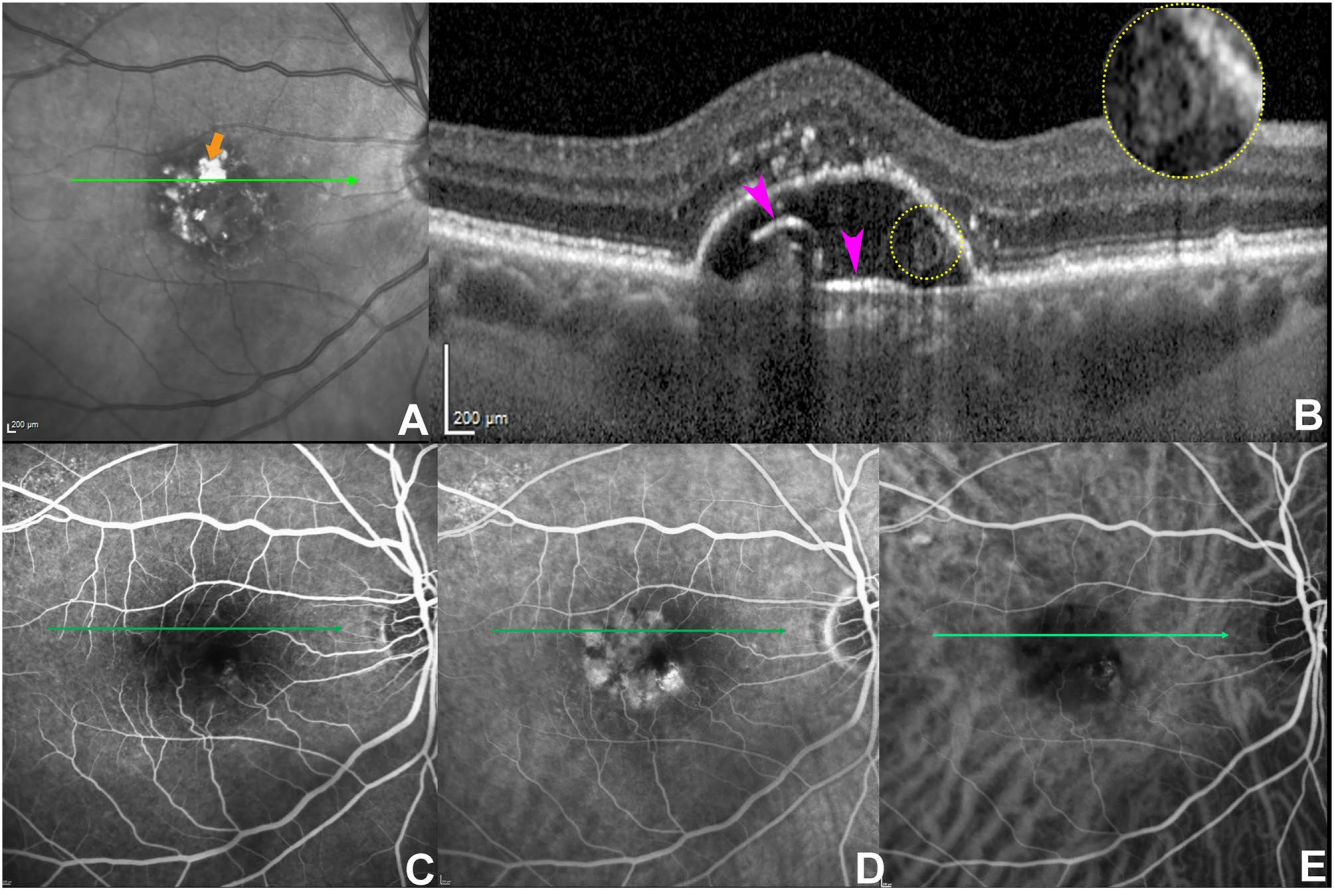
Anatomical variation of sub-retinal pigment epithelium (RPE) tubules. (**A**) Near-infrared reflectance with the green line indicating the location of the B-scan. The orange arrow indicates a large hyperreflective plaque representing hyperreflective crystalline deposits (HCD). (**B**) Optical coherence tomography B-scan showing the presence of HCD (purple arrowheads) in the sub- RPE space parallel to Bruch's membrane. The circled yellow dots indicate the presence of a sub-RPE tubulation attached to either basal lamina or basal laminar deposits. The lesion is not discernable on both fluorescein angiography (FA) and indocyanine green angiography (ICGA) (**C**) FA at 0:41 s obtained at the same visit. (**D**) Late FA acquired at 5 min and 6 s; (**E**) ICGA at 1 min and 6 s.

On OCT B-scans, the integrity of the RPE layer atop appeared to be preserved in 5/7 of cases (71.4%), the presence of overlying HRF was detectable in 4/7 (57.1%). The anatomical position of the SRT appeared to lie above BrM, not floating nor dispersed into the PED material but more likely lodged or anchored to BrM in 5/7 cases (71.4%). Still, in 2/7 cases (28.6%), SRT was separated from BrM (Figs. 2 and 3). Specifically, in one case, SRT was located in the central dome of the PED, close to the BrM but well separated from it. An additional case demonstrated the lesion attached posteriorly to RPE to either its BL or BLamD (Fig. 3). In this specific case, highly reflective plaques were discernible on NIR corresponding to HCD located anteriorly to BrM on OCT B-scan in correspondence of the sub-RPE-BL tubules. FA and ICGA performed on the same day (Fig. 3) did not show any abnormal filling, blockage, or leakage in correspondence of the SRT. An additional case with completed dye tests demonstrated hyperfluorescence on FA and slightly hyperfluorescence on ICGA in correspondence to the lesion, which was also accompanied by an overlying RPE discontinuity with hypertransmission. OCTA did not show evident flow signal alterations within the lesion in all the cases considered (Fig. 2), see also Table 1.

**Figure 4:**
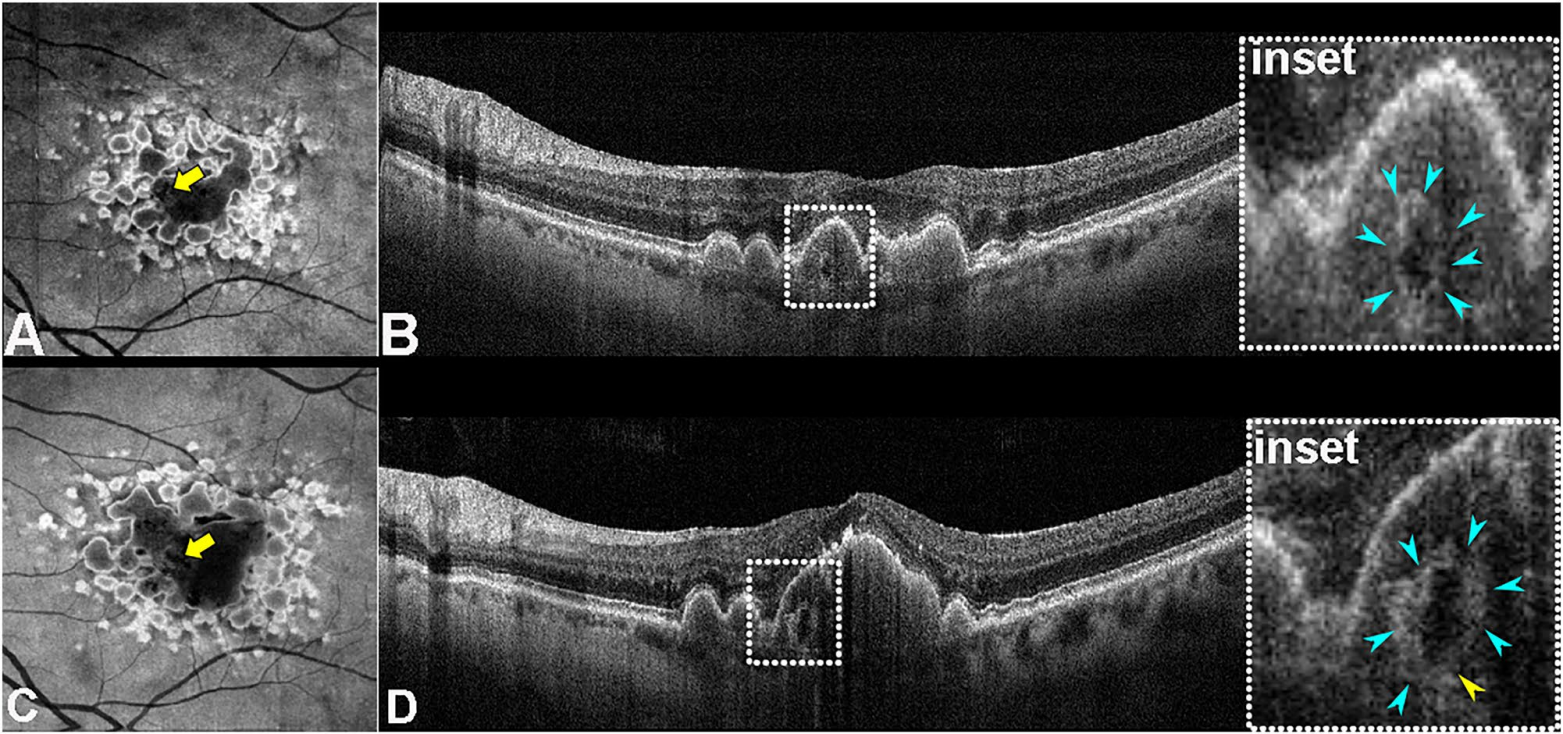
En-face view of the sub-retinal pigment epithelium (sub-RPE) tubules. (**A**) En-face structural optical coherence tomography angiography (OCTA) showing confluent drusen forming a drusenoid pigment epithelium detachment (PED). The lesion can be seen in the context of PED similar to a tube with an hyporeflective interior (yellow arrow). (**B**) Subfoveal OCT b-scan demonstrated a drusenoid PED with an ovoidal structure enclosed. The magnified inset reveals the structure in greater detail (aqua arrowheads) characterized by a hyperreflective border with an hyporeflective interior. This sub-RPE structure, similar to a closed tubulation, seems to be directly in contact with the Bruch's membrane. (**C**) OCTA structural en-face after 4 years showing the tubular appearance (yellow arrow). (**D**) Subfoveal OCT b-scan at 4-year follow-up demonstrating an enlarged drusenoid PED with the sub-RPE tubulation (inset) persistence at the same anatomical location.

Three cases had data available from serial examinations for a mean follow-up time of 5 years (range 5–8 years). The lesions remained stable in number, shape, and reflectivity over time. In a case with longitudinal evaluation, the en-face structural slab showed SRTs as tubular and linear structures within the drusenoid PED, supporting the use of the descriptive terminology ‘tubules’ (Fig. 4). One eye (1/3, 33.3%) developed cRORA with a plateau signature after PED collapse at the end of follow-up (8 years later). The case is described in detail for an optimal interpretation.

### A case evolving into cRORA

**Figure 5:**
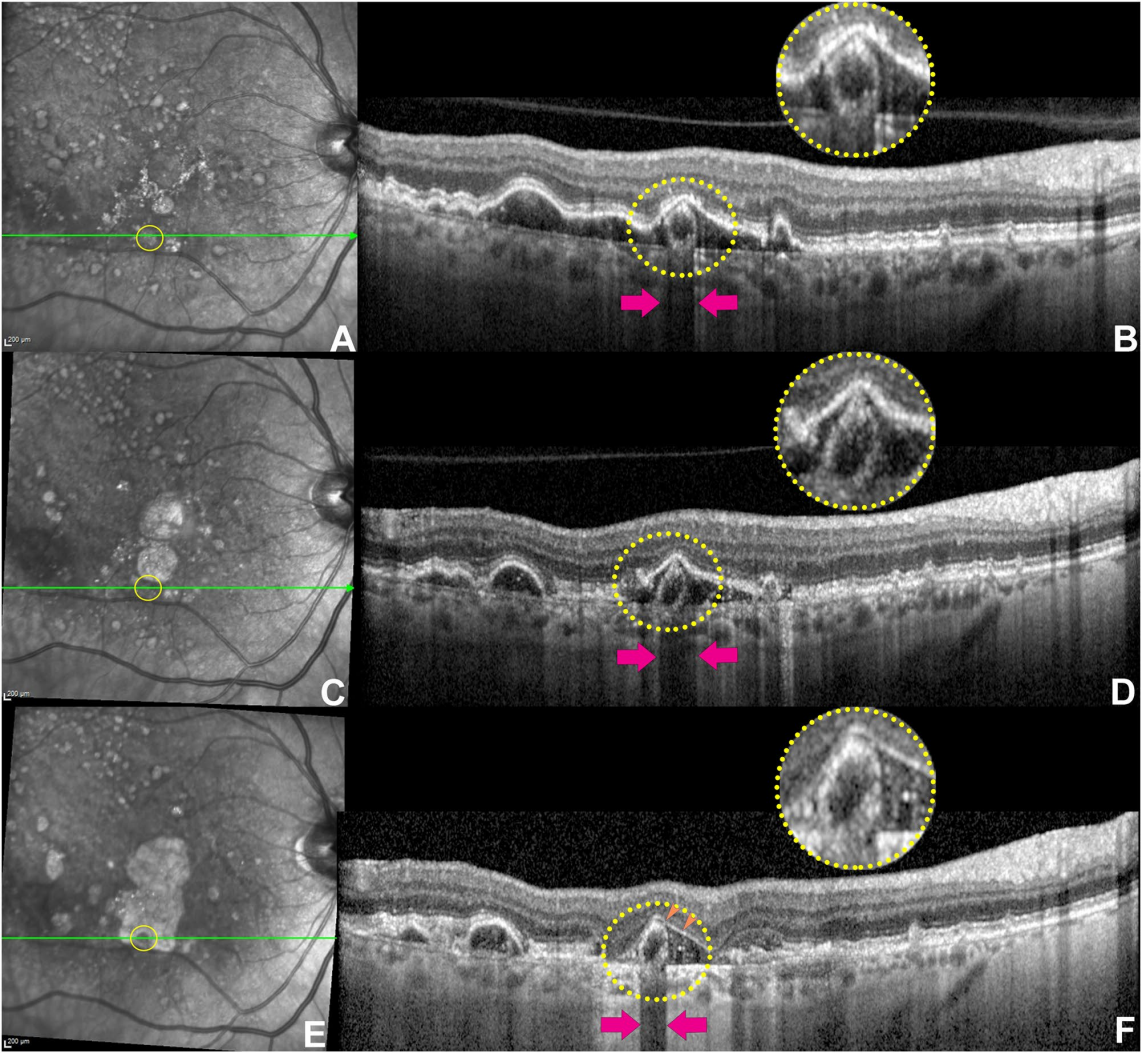
Optical coherence tomography (OCT) sequence of a longitudinal follow-up developing atrophy. (**A**) Near-infrared reflectance (NIR) with the green line indicating the B-scan location and the yellow circle tracking the sub-RPE tubulation (SRT) projection on NIR. (**B**) OCT B-scan at baseline showing a well evident closed SRT between the RPE-BL or BLamD and Bruch's membrane (dotted yellow circle, magnification). The purple arrows indicate a posterior optical shadowing in correspondence of the lesion. (**C**) Tracked NIR after 3 years. (**D**) OCT b-scan demonstrates a progressive drusenoid pigment epithelium (PED) collapse with a persistence of the SRT at the exact same location but slightly tilted laterally. The optical posterior shadowing (purple arrows) is still present. (**E**) Tracked NIR 8-years later. The atrophic changes involved the area in correspondence of the SRT (yellow circle), making it clearly visible as an iso-hypo reflective roundish lesion in contrast with the surrounding hyperreflectivity. (**F**) On OCT b-scan, the PED evolved into a plateau signature after the RPE loss. The plateau characteristically presents a residual thin hyperreflective surface (orange arrowheads) embracing the SRT, which remains stable compared to the follow-up on (**D**) with a well-demarcated posterior shadowing (purple arrows).

A 71-year-old male presented with intermediate AMD and a collapsing PED in the RE. At presentation, BCVA was 20/32 in the RE. On OCT B-scan, a closed SRT was distinctly recognizable in the sub-RPE-BL space of a collapsing drusenoid PED (Fig. 5). The RPE band overlying the lesion appeared to be intact, and optical shadowing into the choroid was associated with the SRT. After 3 years, progressive PED collapse with associated RPE irregularities was noted. However, the SRT persisted at the exact same location but tilted slightly laterally (Fig. 5), and the inferior border appeared fused to the BrM underneath. After 8 years, macular atrophy expanded, as seen on NIR (Fig. 5E), enclosing the SRT. At this point, the presence of atrophy allowed the visualization of the SRT as an iso-hypo reflective roundish lesion in contrast with the surrounding hyperreflectivity on NIR (Fig. 5E). On OCT B-scan, the PED evolved into a plateau signature discernable for the presence of a residual thin hyperreflective surface embedding the SRT and a hyporeflective interior with fine dispersed HRF (Fig. 5F).

**Figure 6:**
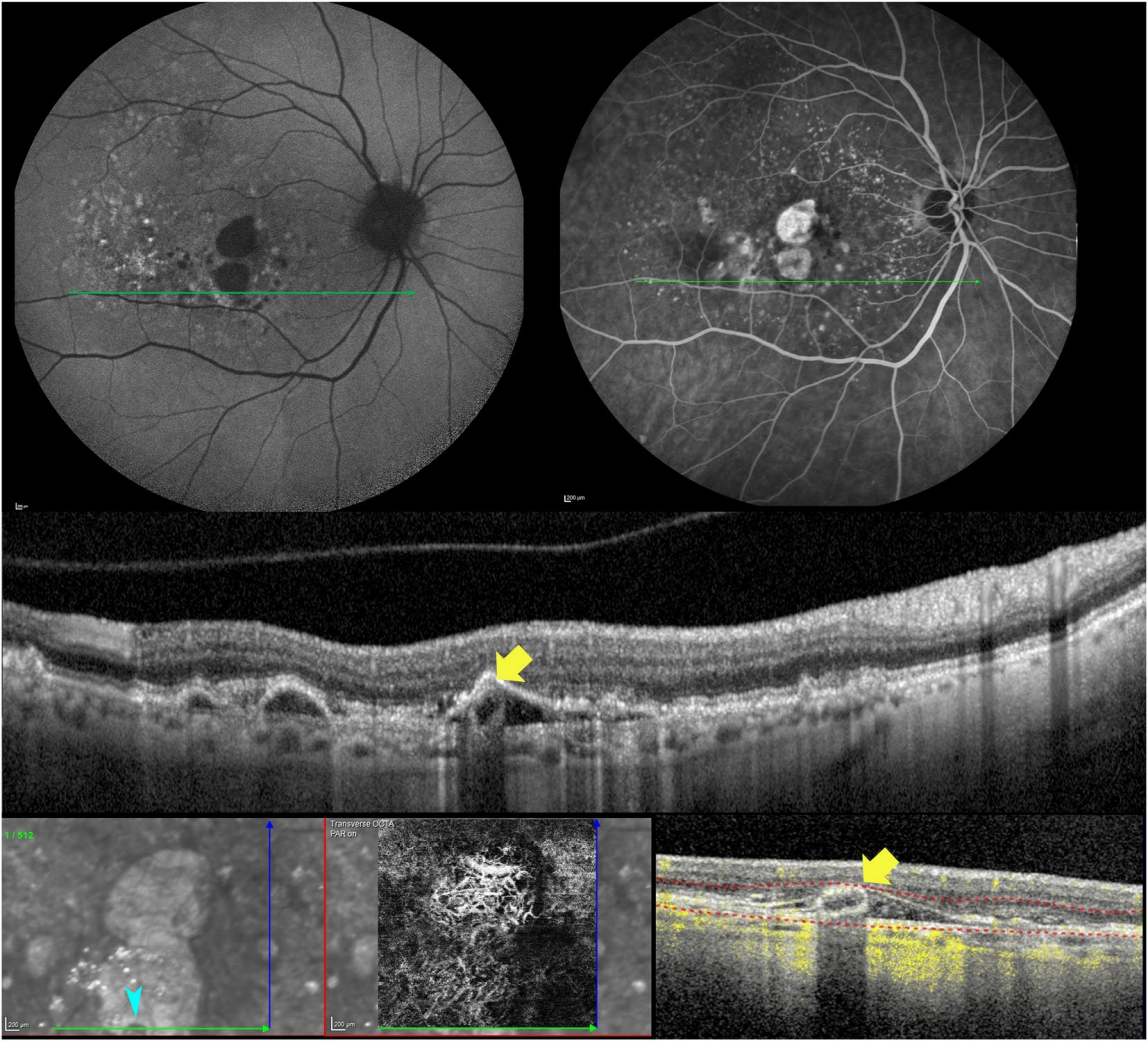
Multimodal imaging of the case with a longitudinal follow-up. (**A**) Fundus autofluorescence performed 4 years after presentation. (**B**) Fluorescein angiography performed on the same day (2.41 min), tracking the lesion through an optical coherence tomography (OCT) B-scan; (**C**) OCT b-scan showing the tubulation located in the sub-RPE-BL space overlying the Bruch's membrane. Optical coherence tomography angiography (OCTA) was not available at this follow-up time. (**D**) OCTA structural en-face 2 years later, the lesion can be discerned in contrast to the surrounding atrophy (aqua arrowheads). (**E**) OCTA avascular slab. (**F**) OCTA b-scan showing the lesion in the context of the plateau that did not show any flow signal.

FAF and FA were available 4 years after the presentation (Fig. 6A,B), demonstrating no frank alterations attributable to the SRT. At this stage, the RPE was still preserved over the SRT lesion (Fig. 6C). OCTA was not available at the same visit, but it was performed 2 years later once the atrophy involved the lesion (Fig. 6D). No evident flow signals were noticed in correspondence with the SRT on both OCTA vascular slabs or OCTA b-scan (Fig. 6E,F).

## Discussion

The present study describes SRT, a novel OCT signature in eyes with non-neovascular AMD. The prevalence of this signature is likely uncommon, but it could not be determined as the population was retrospectively investigated in three different centers with heterogeneous study modalities. As this clinical entity shares a similar OCT appearance with the previously described ORT, we chose to describe our findings as “sub-RPE tubules”. The ovoidal structures described herein were solitary and characterized by a hyperreflective boundary and an hyporeflective interior, exactly like ORT. SRT differed from ORT in topographical localization, number, and relative integrity of the RPE overlying the tubules. Regarding the topographical localization, the SRT was located in the sub-RPE-BL space anteriorly to the BrM. Contrariwise, ORTs were situated invariably within the ONL. Moreover, ORTs can often be multiple formations concomitant with atrophy or fibrosis^8,19^, in contrast with SRT, which were found to be solitary lesions in all the cases. In SRT, there was no evidence of scrolled ELM descent or atrophic changes suggesting an etiology similar to the ORT (Supplementary Fig. S1)^5,9^. In fact, ORT contains degenerated cones pointing towards a lumen encircled by ELM, which is formed with Müller cells. The hyperreflective border of ORT is derived from a combination of ELM and mitochondria of degenerating inner segments^5,8,20^. Müller cells are considered instrumental in forming ORT, but also plateau and corrugations, where the Müller cell processes may extend through the RPE-BLamD complex defects^15^. The change of the sub-RPE material composition within a drusenoid PED may also allow a more easily penetration of these cellular processes^6,15,21^. Because SRTs are localized underneath an intact RPE + BL, the contribution of Müller cells in their formation seems unlikely. Although the Müller cells contribution in SRT formation cannot be excluded, the lack of ELM descent, atrophic changes, and an apparently intact RPE band remained substantial factors against this hypothesis. Taken together, these findings seemed to exclude the same pathogenesis of ORT (Supplementary Fig. S1), pointing towards other possible interpretations.

Outer retinal corrugations have been described on OCT B-scans as curvilinear hyperreflective material above the BrM with the internal border contiguous with the outer portion of the RPE band, believed to represent persistent BLamD^21^. These findings resembled more likely hyperreflective folds seen within atrophic areas far from the hyperreflective ovoidal structures described in our series (Supplementary Fig. S1).

Calcified nodules (Supplementary Fig. S1) are characterized by an accumulation of mineral constituents, mostly hydroxyapatite, within sub-RPE-BL space^2^. HIRD can be identified on OCT as a hyporeflective core encircled by a hyperreflective cap with hyperreflective dots, which may mimic SRT appearance. Unlike SRT, however, this OCT signature is often accompanied by a glistening appearance on color fundus photograph, hyperreflectivity on NIR, and variable FAF manifestations^2,22^. The whitlockite spherules represent the reflective component of refractile drusen, responsible for the glistening and the reflectivity on OCT^2,23^. Despite this, refractile drusen did not show significant light scattering, shadowing, or artifacts on B-scans as appreciable for HCD^17^.

To our knowledge, SRT did not exhibit glistening on CFP, NIR, or hyperreflectivity on FAF, and the well-defined oval hyperreflective structure has been maintained stable over the years without significant changes. A summary of the multimodal features of calcified nodules/HIRD and SRT is shown in Table 2. Data on OCTA, FA, and ICG are not reported for calcified structures and thus not included for comparison. These clinical features and multimodal imaging findings may be crucial for distinguishing between refractile drusen and SRT.

**Table 2:** Clinical and multimodal characteristics of calcified nodules/HIRD and sub-retinal pigment epithelium tubules (SRT).

	Calcified nodules/HIRD	SRT
CFP	Glistening visible after RPE loss (stage 3A)	No-glistening
FAF	Hyper FAF (Stage 2A) Hypo center with hyperreflective border (Stage 3A) Hypo-FAF (Stage 4A)*	Hypoautofluorescent (60%) Isoautofluorescent (40%)
NIR	Hyperreflectivity (all stages)	Hyporeflective (60%) Soreflective (20%) Hyperreflective (20%)
OCT	Hyporeflective area within druse (Stage 2A) Hyperreflective cap with hyporeflective interior, focal RPE loss (Stage 3A) Hyperreflective border and hyporeflective core, complete RPE loss (Stage 4A)	Ovoidal lesions with hyperreflective border and hyporeflective center Loss of RPE in 28.6% with complete RPE loss in a case No evidence of evolutive stages

*CFP* color fundus photograph, *FAF* Fundus autofluorescence, *NIR* Near-infrared reflectance, *OCT* Optical coherence tomography, *RPE* retinal pigment epithelium.

*Stage 4A is characterized by complete RPE loss with a thin residual veil of basal laminar deposits.

Two variations, A and B, with the same four-stage progression system were proposed^2^. The variation B differed for the presence of a thicker BLamD cap with less refractility on CFP, increased hyper-FAF, and less reflectivity on NIR. Therefore, it may be possible that SRTs are calcified variants with less refractility similar to variant B, but the FAF appearance, the reflectivity on NIR, and the lack of staging or evolutionary changes are against this hypothesis. A possible explanation may involve the combination of these sub-RPE calcific deposits with other components, as reported by Thompson et al.^24^. The authors hypothesized that the sub-RPE hydroxyapatite spherules can bind different proteins, along with lipid deposition and entrapment, leading to a self-driven oligomerization and growth of the deposits reaching noticeably size. In this regard, the DPED microstructure comprised of lipoprotein-derived debris and mixed lipid pools^14^ may offer a substrate for nucleation and growth of the calcific deposits.

Noteworthy, clinicopathologic and histopathologic studies demonstrated calcific nodules within drusenoid PED associated with subretinal and intraretinal migration of RPE cells^14,25,26^. Ex vivo correlations between spectral-domain OCT and high-resolution histologic photomicrographs revealed small refractile spherules of calcium phosphate and large refractile nodules occupying the central dome of PED corresponding to punctate hyperreflective foci on OCT B scan^14^. Although documented ex vivo, the anatomical localization and the OCT findings significantly differ from the SRTs description, perhaps indicating a different conformational state but also leaving open other potential explanations for our findings.

Human RPE cells contain abundant organelles, including lipofuscin, melanolipofuscin, melanosomes, and mitochondria. The RPE morphology, orientation, and the organelles distribution contribute to autofluorescence signal on FAF and the reflectivity on OCT^6,27,28^. The organelles (i.e., melanosomes, lipofuscin, melanolipofuscin, and mitochondria) produce the intense OCT signal and posterior optical shadowing from the RPE layer with a minimal scattering from the nuclei^27–29^. These optical characteristics support the hypothesis of a tubulation composed of RPE cells, as shown in the illustrative histological case (Fig. 6). Further corroborating this hypothesis, SRT showed an intense posterior optical shadowing on OCT in contrast with the surrounding and diffuse hypertransmission due to RPE loss. In a case with longitudinal follow-up developing cRORA, the reflectivity in the area occupied by the SRT remained iso-hyporeflective on NIR in comparison with the dense hyperreflectivity caused by a diffuse RPE atrophy (Fig. 5). On NIR, the lesion can only be visualized with the loss of the overlying RPE band, and the resulting roundish alteration presented the same reflectivity of the background with intact RPE. On B-scan, SRT maintains the ovoidal shape with hypereflective contour and hyporeflective interior and an intense posterior shadowing.

From previous histopathological evidence, it has been demonstrated that RPE cells may transdifferentiate into a migratory phenotype and migrate through the ELM into the retina. A phenotype of subducted cells may migrate between BLamD and BrM, originating from dissociated RPE cells that migrate horizontally from atrophy to a less affected area^1,6,27,30^. RPE cells undergoing epithelial-mesenchyme transition (EMT) might resist cell death, remaining dysfunctional. One of the main factors promoting EMT is hypoxia, which is a condition common with the distancing of RPE from choriocapillaris, especially in a PED. As previously suggested, RPE migration can be toward retinal vessels in the deep capillary plexus^1,14^ along BrM as subducted cells to find oxygen and nutrients^6,31^. RPE-BL thickening over a PED usually preceded the RPE cells sloughing and migration^14,27^. Therefore, one additional hypothesis on SRT origin can include clustering activated RPE cells with some residual BLamD, attracted to the BrM driving for oxygen. An illustrative histological case provided by human donors obtained from a research eye collection at the University of Iowa demonstrates the ability of RPE cells to reduplicate and organize to form tubular structures made entirely of RPE, which continue to form monolayers (Supplementary Fig. S2)^32,33^.

Clinical and prognostic implications of sub-RPE-BL tubules include the distinction with neovascularization growing into the PED, particularly polypoidal or aneurysmal forms. Indeed, in such cases, the appearance of a hyporeflective round sub-RPE structure may suggest the presence of a dilated vessel or aneurysm. According to the proposed consensus nomenclature for polypoidal lesions, the sub-RPE ring-like signature appears as a round structure with hyperreflective contour and hyporeflective core that may vary in reflectivity and thus very similar to the SRT^34^. SRT structures are benign, solitary lesions that remain stable in number, shape, and reflectivity over the years in the absence of neovascularization. Also, the appearance of PED associated with SRT denotes a dome-shaped RPE elevation measuring at least 350 µm in the narrowest diameter, in contraposition with sharp-peaked PED with inverted “V” configuration, sometimes described as a ‘thumb-like protrusion” typically associated with polypoidal lesions.

Another interesting point for future studies is represented by the determination of whether SRT lesions might be associated with DPED collapse and atrophic changes.

Limitations of the present study are the limited series of cases described, the lack of a direct clinicopathological correlation, and its retrospective nature. Nevertheless, this study offered a novel observation that may encourage further clinical and histopathological studies to characterize this OCT signature better. The present study is an observational cross-sectional design with a lower evidence level. Thus, further case–control or cohort studies with appropriate statistical power should be performed better to understand the clinical and prognostic significance of SRT.

In conclusion, sub-RPE-BL tubules represent an OCT signature characterized by an ovoidal sub-RPE-BL or BLamD structure with hyporeflective lumen and delineated by a hyperreflective wall associated with drusenoid PED in non-neovascular AMD. Their multimodal imaging appearance shares some similarities with calcified structures, but some discrepancies may suggest a different genesis. An alternative hypothesis may include a possible RPE origin, but it needs to be determined. The multimodal findings are particularly helpful in making an accurate differentiation with other existing OCT signatures. Further studies would be necessary to understand the potential prognostic implications of SRTs in the drusenoid PED lifecycle.

## Supplementary Information

Supplementary Figure S1.

Supplementary Figure S2.

## Data Availability

Data and images are available upon request to corresponding author Giuseppe Querques.
