# Supplementary material for: Sub-retinal pigment epithelium tubules in non-neovascular age-related macular degeneration
Source: Sci Rep. 2022 Sep 7;12:15198. doi: 10.1038/s41598-022-19193-6 (PMC9452588; doi:10.1038/s41598-022-19193-6)
Supplement: Supplementary file 1 — Supplementary Figure S1. [file 41598_2022_19193_MOESM1_ESM.pdf]

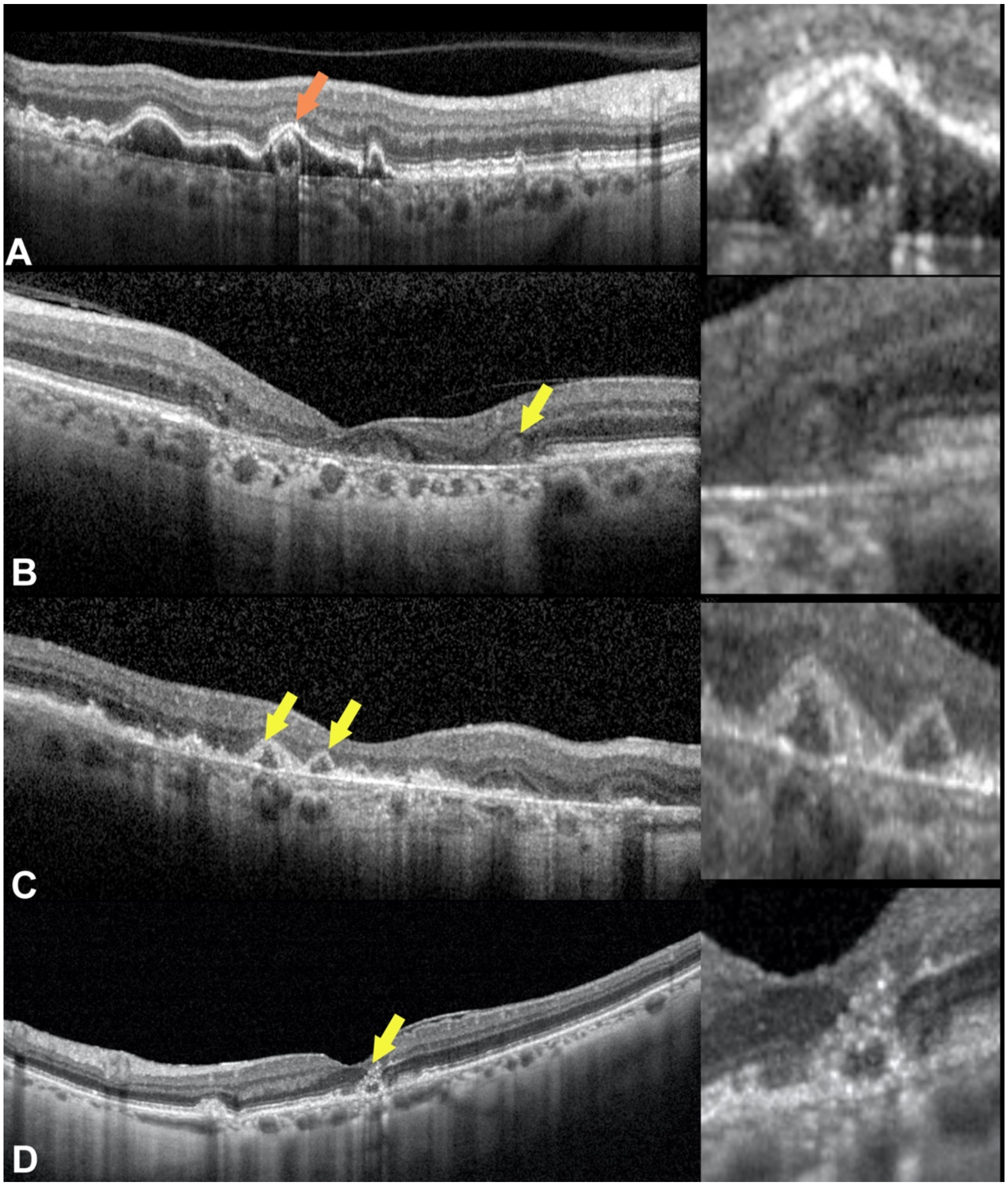

Supplementary Figure S1. Spectral-domain optical coherence tomography B-scans showing differential diagnosis. **A.** Sub-retinal pigment epithelium (RPE) tubulation (orange arrow), magnification inset (right). **B.** Outer retinal tubulation (ORT) (yellow arrow), magnification inset (right). **C.** Outer retinal corrugations (yellow arrows), magnification inset (right). **D.** Refractile drusen/ heterogeneous internal reflectivity within drusen (HIRD) (yellow arrow), magnification inset (right).
