# Supplementary material for: Sub-retinal pigment epithelium tubules in non-neovascular age-related macular degeneration
Source: Sci Rep. 2022 Sep 7;12:15198. doi: 10.1038/s41598-022-19193-6 (PMC9452588; doi:10.1038/s41598-022-19193-6)
Supplement: Supplementary file 2 — Supplementary Figure S2. [file 41598_2022_19193_MOESM2_ESM.pdf]

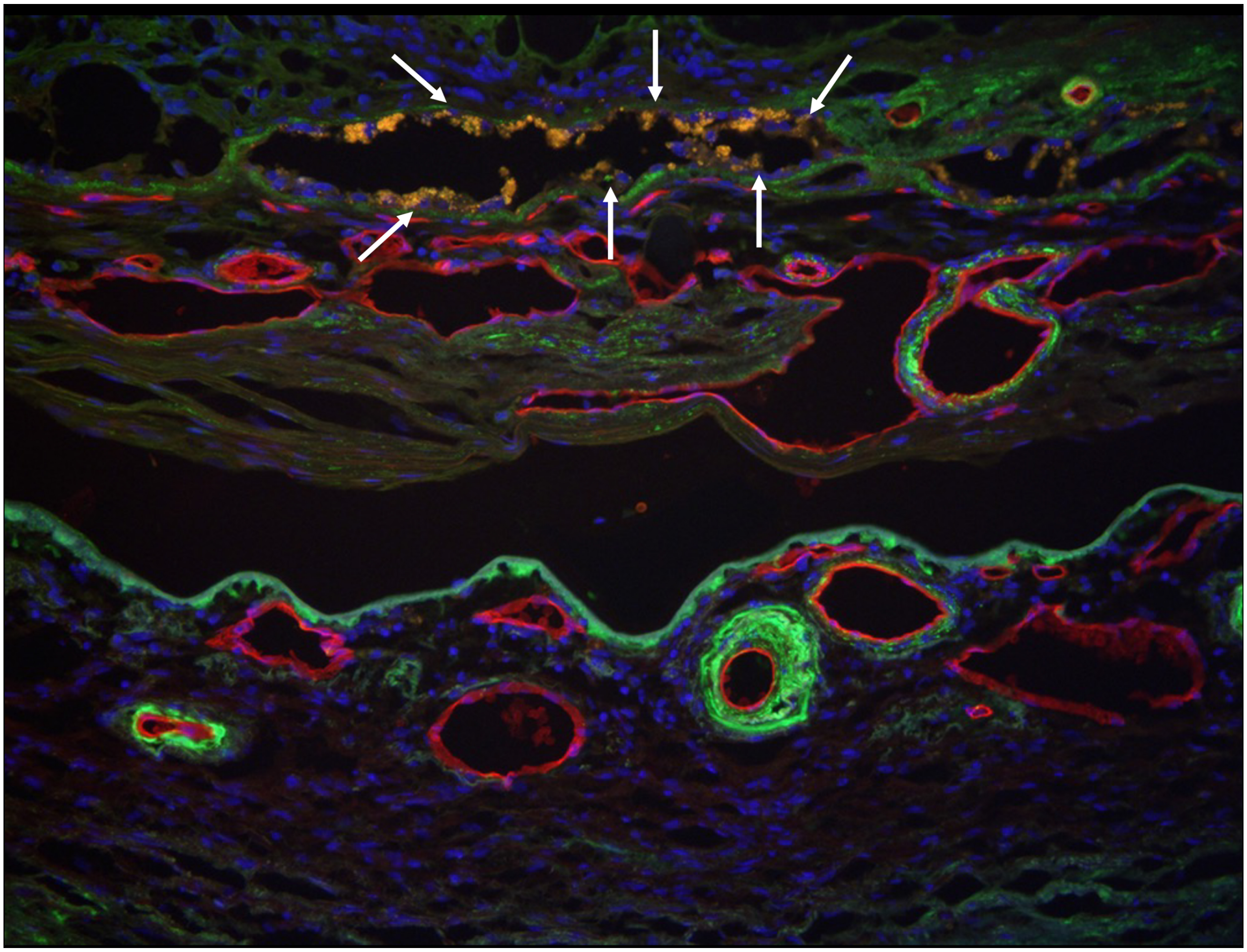

Supplementary Figure S2. An illustrative histological case from an 83-year-old female with choroidal neovascularization in the right eye. The sample was preserved in 4% paraformaldehyde in 1X phosphate buffered saline, pH 7.4, within 9 hours after death. After 2 hours fixation, the specimens were washed in phosphate-buffered saline and then were cryoprotected in sucrose and embedded in sucrose-optimal cutting temperature medium, as previously described by Barthel and Raymond.[32] Tissue sections were collected on a cryostat and dual-labeled with anti-C9 neoepitope antibody (green) and the vascular-labeling lectin UEA-I (red). A neoepitope present in activated complement C9 that is exposed during formation of the MAC (anti-C5b-9 complex;

Dako, Carpinteria, CA) and the vascular marker Ulex europaeus agglutinin I (UEA-I; Vector Laboratories, Burlingame, CA), which labels fucosylated glycoconjugates on viable human endothelial cells, were employed.[33] MAC and endothelial cells were detected using Alexa Fluor 488-conjugated goat anti-mouse IgG (Life Technologies, Carlsbad, CA) and avidin-Texas Red (Vector Laboratories), respectively In the context of the neovascularization, a reduplicated retinal pigment epithelium (RPE) cells forming an island (white arrows) composed of RPE cells with characteristic yellow autofluorescence. Labeling with antibodies directed against MAC is predominantly surrounding vessels and in Bruch's membrane, which shows striking loss of choriocapillaris.
